# General Commentary: Synovial sarcoma of the head and neck: A review of reported cases on the clinical characteristics and treatment methods

**DOI:** 10.3389/fcell.2023.1164523

**Published:** 2023-03-03

**Authors:** Jiro Ichikawa, Hiroki Imada, Satoshi Kanno, Tomonori Kawasaki

**Affiliations:** ^1^ Department of Orthopaedic Surgery, Interdisciplinary Graduate School of Medicine, University of Yamanashi, Yamanashi, Japan; ^2^ Department of Pathology, Saitama Medical Center, Saitama Medical University, Kawagoe, Saitama, Japan; ^3^ Department of Pathology, Saitama Medical University International Medical Center, Saitama, Japan

**Keywords:** synovial sarcoma, immunohistochemistry, adjuvant therapy, primary site, head and neck, rare case

## 1 Introduction

We read with interest the article by Dr. Quan and others entitled: “Synovial sarcoma of the head and neck: A review of reported cases on the clinical characteristics and treatment methods,” published in *Frontiers in Cell and Developmental Biology* (Quan et al., 2022). The authors reviewed many case reports in which primary synovial sarcomas (SS) of the head and neck are described. However, the clinical and pathological information provided in those reports was insufficient. Although every case report of such a rare disease is valuable, case eligibility should be carefully considered for such reviews if one truly wants to understand the clinicopathological characteristics of SS of the head and neck compared with those of SS of the extremities. We discuss our reasoning below.

## 2 Subsections relevant for the subject

### 2.1 Eligibility for case series of SS

SS is a high-grade sarcoma accounting for 5%–10% of all soft-tissue sarcomas (Kransdorf, 1995), with the SS18–SSX fusion gene observed in >95% of SS cases (Saito, 2013). The most frequent sites of SS are the soft tissue of the lower and upper extremities (70% of cases), trunk (15%), and head and neck (7%) (Suurmeijer et al., 2020). Bone is considered an unusual site for SS, seemingly rarer than the head and neck (Suurmeijer et al., 2020). Recently, Righi et al. (2022) reported on 430 cases that had previously been diagnosed as spindle-cell sarcoma of the bone, 25 of which, upon retrospective analysis, were diagnosed as primary SS of the bone. The inclusion criteria were as follows: i) diagnosis of SS of the bone, molecularly confirmed using fluorescence *in situ* hybridization (FISH) and reverse transcription–polymerase chain reaction and/or immunohistochemical (IHC) analysis; ii) availability of histopathology, imaging, and clinical information; iii) exclusion of the possibility of bone metastasis (Righi et al., 2022). These criteria are the main focus of our concerns about the review article by Quan et al. (2022).

## 3 Discussion

### 3.1 The role of immunohistochemistry in diagnosis

In terms of IHC, cytokeratin, EMA, CD99, and TLE1 are positive markers of SS, while CD34, S-100, and MDM2 are negative markers (Thway and Fisher, 2014). The former are also positive markers in the case of other tumors (Thway and Fisher, 2014). Hence, the diagnosis of SS *via* IHC is not clear cut, and it is often combined with FISH or PCR. To overcome the limitations of IHC, Zaborowski et al. (2020) reported the efficacy of SS18–SSX and SSX antibodies in the diagnosis of SS. Surprisingly, the sensitivity and specificity of SS18–SSX antibodies were 87.2% and 100%, respectively, and those of SSX antibodies were 92.3% and 96.1%, respectively (Zaborowski et al., 2020). Nevertheless, caution is required, as i) handling of bone samples leads to a reduction in antigenicity owing to decalcification, and ii) false-positive results with SSX antibodies have been reported for other sarcomas (Zaborowski et al., 2020; Righi et al., 2022). Even accounting for the above, SS18–SSX and SSX antibodies seem to have similar accuracy to FISH or PCR. In the review by Quan et al. (2022), several diagnoses were made without information, not only of FISH or PCR, but even of IHC. Such cases should have been excluded from the review because they might have been misdiagnosed.

### 3.2 Clinical and imaging-based information

Once SS has been detected in the head and neck, clinicians should determine whether it is primary or a metastasis. If primary, they should perform imaging to detect any metastases. In fact, cervical metastasis of SS of the head and neck has been reported (Ishiki et al., 2009). Determination of the primary site in the case of SS is challenging (Ichikawa et al., 2022). Positron emission tomography–computed tomography or computed tomography (CT) performed to exclude other tumors may be used to determine whether the primary site is the head and neck. Considering these points, one case in which only biopsy results were reported would have had to be excluded from the review by Quan et al. (2022). Although characteristic imaging features of SS have been reported, including calcification (*via* CT) and the triple sign and pseudo-cystic sign (*via* magnetic resonance imaging), those results had relatively low sensitivity (Tordjman et al., 2023). However, those imaging features were useful for surgical planning. Larger tumors are correlated to a worse prognosis of SS of the extremities (Hagiwara et al., 2023). Thus, appropriate information in terms of imaging features will enable a better understanding of the characteristics of SS of the head and neck.

### 3.3 Adjuvant therapy

In general, wide resection margins and adjuvant chemotherapy are the preferred treatment for SS of the extremities (Hagiwara et al., 2023). Chemotherapy use reportedly correlates not only with better overall and disease-free survival among patients with SS of the extremities (Hagiwara et al., 2023), but also with better 10-year disease-free survival among patients with primary bone SS (Righi et al., 2022). Ifosfamide and doxorubicin have been validated as first-line treatment (Spurrell et al., 2005). Although chemotherapy for SS of the head and neck was described in certain cases, details of the administered drugs were generally unclear in cases included in the review by Quan et al. (2022). Standardized regimens for SS of the head and neck have not been established and will require prospective research. The frequent use of radiotherapy for SS of the head and neck (Quan et al., 2022) may be owing to such tumors being located in the vicinity of important vessels, nerves, and organs, being therefore difficult to resect with wide margins. We suggest that future potential studies be focused on the efficacy of radiation in the treatment of SS of the head and neck.

In conclusion, despite the rarity of cases of SS of the head and neck, reviews thereof should use strict eligibility criteria such as: i) precise diagnosis, ii) adequate clinical information, and iii) exclusion of metastasis. Such reviews would enable clarification of the real clinicopathological characteristics of the disease and, in turn, lead to meaningful prospective studies for standardization of therapy.
